# Harnessing Social Networks along with Consumer-Driven Electronic Communication Technologies to Identify and Engage Members of 'Hard-to-Reach' Populations: A Methodological Case Report

**DOI:** 10.1186/1471-2288-10-8

**Published:** 2010-01-20

**Authors:** Melanie J Rock

**Affiliations:** 1 Population Health Intervention Research Centre, University of Calgary, Calgary, Canada; 2 Department of Community Health Sciences, Faculty of Medicine, University of Calgary, Calgary, Canada; 3 Department of Ecosystem and Public Health, Faculty of Veterinary Medicine, University of Calgary, Calgary, Canada; 4 Calgary Institute of Population and Public Health, Calgary, Canada

## Abstract

**Background:**

Sampling in the absence of accurate or comprehensive information routinely poses logistical, ethical, and resource allocation challenges in social science, clinical, epidemiological, health service and population health research. These challenges are compounded if few members of a target population know each other or regularly interact. This paper reports on the sampling methods adopted in ethnographic case study research with a 'hard-to-reach' population.

**Methods:**

To identify and engage a small yet diverse sample of people who met an unusual set of criteria (i.e., pet owners who had been treating cats or dogs for diabetes), four sampling strategies were used. First, copies of a recruitment letter were posted in pet-friendly places. Second, information about the study was diffused throughout the study period via word of mouth. Third, the lead investigator personally sent the recruitment letter via email to a pet owner, who then circulated the information to others, and so on. Fourth, veterinarians were enlisted to refer people who had diabetic pets. The second, third and fourth strategies rely on social networks and represent forms of chain referral sampling.

**Results:**

Chain referral sampling via email proved to be the most efficient and effective, yielding a small yet diverse group of respondents within one month, and at negligible cost.

**Conclusions:**

The widespread popularity of electronic communication technologies offers new methodological opportunities for researchers seeking to recruit from hard-to-reach populations.

## Background

Many populations of interest to health researchers can be hard to reach. These populations may contain few members, be scattered over a large geographic area, be stigmatized, or represent elites with little interest in being studied [1]. The challenges of sampling from hard-to-reach populations are compounded in the absence of accurate or comprehensive information, and whenever few members of a target population regularly interact or even know each other [2]. This paper reports on the sampling strategies deployed for an ethnographic case study that, to explore connections between pet care and human health, focused on pet owner's involvement in treating dogs and cats for diabetes [3,4].

A large number of people worldwide are treating dogs and cats for diabetes, yet I faced logistical, ethical, and resource allocation challenges in recruiting even a small number of them. People with diabetic pets constitute a low-frequency population whose members are geographically disperse and largely nonassociative, meaning that few members know each other personally and that no registry exists [2]. Nevertheless, diabetes is a condition commonly seen in veterinary practice with cats and dogs. Current research suggests that approximately 1 in 500 dogs and 1 in 250 cats are diabetic [5,6]. In this paper, I describe and reflect on my sampling strategies, and in particular, on the power of electronic communication technologies in social networks. The lessons learned may have broad relevance for social and health researchers, given the broad-based popularity of consumer-driven electronic communication.

## Methods

### Recruitment Objectives

This study revolved around ethnographic interviews [7]. An *a priori *recruitment target was set at 10 pet owners who were actively treating a dog or cat for diabetes, or whose diabetic dog or cat had recently died. Given the methodological emphasis on exploration [7,8], I needed to recruit at least 5 pet owners from the local area so as to permit extended face-to-face interviews, to enable *in situ *observation, and to facilitate longitudinal follow-up. (Some interviews with pet owners could be - and were - conducted by telephone [9].) Furthermore, I needed to recruit pet owners whose living situations and socioeconomic circumstances differed sufficiently to provide the basis for illuminating comparisons. In other words, I needed to recruit what is sometimes called a criterion-based intensity sample [10].

### Recruitment Strategies

This study relied on four strategies to recruit pet owners. First, copies of a recruitment letter were posted in a large local park where dogs are permitted off-leash, as well as in two nearby cafés. I had previously noted that a variety of pet-related notices are regularly posted in these locations. Second, information about the study was diffused throughout the study period via word of mouth. This 'organic' strategy was used throughout the study period and involved the lead investigator and other team members casually discussing the project with colleagues, family members and friends, to let them know that I wanted to recruit people who had been treating a cat or dog for diabetes. Third, I personally sent the recruitment letter via email to a friend who owns both a dog and a cat. Fourth, veterinarians were enlisted to refer owners of diabetic pets, including owners who themselves had diabetes. All but the strategy of posting information in public spaces represent forms of *chain referral sampling *[1], in which success hinges on assistance from members of the population of interest or their associates or both, in the context of social networks.

### Ethical Approval

The Health Research Ethics Board at the University of Calgary approved this study, subsequent to successful peer review of the protocol under the auspices of the Social Sciences and Humanities Research Council of Canada's Standard Grants Program. As financial or other incentives were not proffered, participation was strictly voluntary.

## Results

Recruitment of pet owners took place in two phases. In the first phase, I aimed to recruit any pet owners whom I could find with current or recent experience in treating a dog or cat for diabetes. Three strategies were deployed in the first phase: posting a recruitment letter in dog-friendly places (yield = 0 referrals), diffusing information via word-of-mouth through personal and professional networks (yield = 1 referral), and circulating a recruitment letter via email (yield = 11 referrals). In other words, I met my recruitment target with the email strategy alone.

As an ensemble, the twelve pet owners recruited in phase 1 represented a diverse group. They resided in neighbourhoods that were geographically dispersed and that varied in socioeconomic composition. For instance, four lived in neighbourhoods in which the dominant educational attainment was less than high school completion, while another four lived in neighbourhoods in which the dominant educational attainment was at a university level. Neighbourhood median household income ranged from CAD$25-49K (4 participants) to over CAD$100K (1 participant). Nine lived within Calgary city limits. Five were men and seven were women, and all twelve had treated a dog or cat for diabetes over the course of at least one year. Only one participating pet owner was recruited by another. To the best of my knowledge, the other recruits did not know each another.

The email strategy emerged in the context of an informal discussion about the project with a personal friend, who offered to forward the recruitment letter via email to an associate known to be an 'animal lover.' On 9 March 2006, I emailed the letter to my friend, and she subsequently forwarded it to her associate, who in turn forwarded it to others in her network, and so on. In less than a month, I recruited 11 pet owners as a result of this one email message. Notably, only five of these owners personally received the email message. Five found out about the study from a friend, relative, or neighbour, while one was recruited after someone posted the recruitment letter at her dog's daycare.

The second phase of recruitment began approximately nine months after the first. Based on analysis of the interview data from the first phase, which highlighted that the period surrounding the diabetes diagnosis was often tumultuous for owners [3], I sought to recruit at least one pet owner during the diagnostic process. Also, as the first phase had included a pet owner with type 2 diabetes, but no one with type 1 diabetes, I wanted to recruit a pet owner with type 1 diabetes. I sent a letter to the existing sample, thanking them and inviting them to refer additional participants (yield = 0) and I engaged veterinarians to assist with recruiting (yield = 3). Concretely, I followed up with veterinarians whom I had previously interviewed as part of this project [3], I presented results from phase 1 at veterinary rounds convened for professional development and teaching purposes, and I met and corresponded with the business manager for a consortium of veterinary clinics, who in turn circulated information about the study through word-of-mouth and via email.

## Discussion

All of the pet owners who ultimately participated in this study were recruited through some of form of chain referral sampling, which in turn pivoted on word-of-mouth diffusion of information. Chain referrals via email proved to be efficient and effective, yielding a diverse group of respondents within a short time period, and at negligible cost. Using email to assist with recruitment was a novel adaptation of chain referral sampling, which is how researchers have generally succeeded in recruiting from hard-to-reach populations [1,2,11]. In the *snowball sampling *variant of this approach, researchers begin by recruiting one or two people in the population of interest, through key informants or documents, and then asking these recruits to list others who meet the inclusion criteria and then to recommend someone from that list to interview. Similar to snowball sampling, *respondent-driven sampling *begins with recruiting a few people to act as 'seeds'. In contrast to snowball sampling, however, these initial recruits receive compensation if they recruit members from their respective networks. Rather than listing names, the existing recruits distribute coupons. Whenever a new recruit returns one of these coupons, receiving compensation for doing so, the research team use their records to determine which existing recruit had distributed that particular coupon [12].

Using email to adapt the chain referral sampling approach allowed me to tap into many different social networks, stemming from a single 'seed' who did not even belong to the target population. Similar to respondent-driven sampling but unlike snowball sampling, I circumvented the ethical issue of asking for names and contact information without prior consent. While I did not pay recruits or recruiters, recruitment and referral incentives could be incorporated in future studies seeking to harness consumer-driven electronic communication in social networks. Also, while I relied on only one email 'seed' because the initial recruitment objectives were quickly satisfied, future studies with more ambitious recruitment targets could involve multiple email 'seeds.' Also, future studies might deploy multiple consumer-driven communication technologies (e.g., email as well as Facebook^© ^and texting with mobile telephones). In addition, recruitment from hard-to-reach populations could be enhanced through media coverage [13], given that many media stories can now be readily circulated via email. Media coverage posted on-line, in other words, could 'plant' numerous recruitment 'seeds'.

As with respondent-driven sampling, learning about a study through an existing network may have encouraged potential participants to come forward. Yet in this adaptation using a consumer-driven electronic communication technology, most recruiters did not belong to the hard-to-reach population. Put another way, harnessing consumer-driven communication technologies may allow for sampling of sub-populations to occur through appeals to a broader population: fully half of the people recruited via email did not personally receive the recruitment letter. The positive results reported in this paper may, however, reflect the 'human interest' angle and novelty of pets.

## Conclusions

Consumer-driven electronic communication has become ubiquitous in many contexts worldwide. To the extent that such communication technologies increasingly constitute part of the fabric of social life, researchers may sometimes wish to tap them when seeking to sample from hard-to-reach populations.

## Competing interests

The authors declare that they have no competing interests.

## Authors' contributions

Melanie Rock designed the study upon which this paper is based and wrote the manuscript.

## Pre-publication history

The pre-publication history for this paper can be accessed here:

http://www.biomedcentral.com/1471-2288/10/8/prepub
